# Acute Radiation Colitis after Preoperative Short-Course Radiotherapy for Rectal Cancer: A Morphological, Immunohistochemical and Genetic Study

**DOI:** 10.3390/cancers12092571

**Published:** 2020-09-09

**Authors:** Magda Zanelli, Alessia Ciarrocchi, Giovanni De Petris, Maurizio Zizzo, Massimo Costantini, Alessandra Bisagni, Federica Torricelli, Davide Nicoli, Dafne Ramundo, Stefano Ricci, Andrea Palicelli, Francesca Sanguedolce, Stefano Ascani, Carolina Castro Ruiz, Valerio Annessi, Raffaella Zamponi, Mara Bortesi, Veronica Martino, Marialisa Marchetti, Loredana De Marco

**Affiliations:** 1 Pathology Unit, Azienda Unità Sanitaria Locale-IRCCS di Reggio Emilia, 42122 Reggio Emilia, Italy; Magda.Zanelli@ausl.re.it (M.Z.); Alessandra.Bisagni@ausl.re.it (A.B.); Stefano.Ricci@ausl.re.it (S.R.); Andrea.Palicelli@ausl.re.it (A.P.); Mara.Bortesi@ausl.re.it (M.B.); Veronica.Martino@ausl.re.it (V.M.); Marialisa.Marchetti@ausl.re.it (M.M.); Loredana.DeMarco@ausl.re.it (L.D.M.); 2 Laboratory of Translational Research, Azienda Unità Sanitaria Locale-IRCCS di Reggio Emilia, 42122 Reggio Emilia, Italy; Alessia.Ciarrocchi@ausl.re.it (A.C.); Federica.Torricelli@ausl.re.it (F.T.); 3 Dianon Pathology, Shelton, CT 06484, USA; depetrg@labcorp.com; 4 Surgical Oncology Unit, Azienda Unità Sanitaria Locale-IRCCS di Reggio Emilia, 42122 Reggio Emilia, Italy; Carolina.CastroRuiz@ausl.re.it (C.C.R.); Valerio.Annessi@ausl.re.it (V.A.); 5 Clinical and Experimental Medicine PhD Program, University of Modena and Reggio Emilia, 41121 Modena, Italy; 6 Scientific Director, Azienda Unità Sanitaria Locale-IRCCS di Reggio Emilia, 42122 Reggio Emilia, Italy; Massimo.Costantini@ausl.re.it; 7 Laboratory of Molecular Biology, Azienda Unità Sanitaria Locale-IRCCS di Reggio Emilia, 42122 Reggio Emilia, Italy; Davide.Nicoli@ausl.re.it (D.N.); Raffaella.Zamponi@ausl.re.it (R.Z.); 8 Radiotherapy Unit, Azienda Unità Sanitaria Locale-IRCCS di Reggio Emilia, 42122 Reggio Emilia, Italy; Dafne.Ramundo@ausl.re.it; 9 Pathology Unit, Azienda Ospedaliero-Universitaria-Ospedali Riuniti di Foggia, 71122 Foggia, Italy; fsanguedolce@ospedaliriunitifoggia.it; 10 Pathology Unit, Azienda Ospedaliera S. Maria di Terni, University of Perugia, 05100 Terni, Italy; s.ascani@aospterni.it

**Keywords:** neoadjuvant, radiation, radiotherapy, short-course, long-course, rectal cancer, colitis

## Abstract

**Simple Summary:**

Radiotherapy is the standard treatment for locally advanced rectal cancer with a high risk of local recurrence, if treated with surgery alone. The two regimens accepted are preoperative long-course radiotherapy with concomitant chemotherapy and preoperative short-course radiotherapy. The aim of our retrospective study is to provide a comprehensive morphological description of radiation-induced changes in rectal cancer specimens. We compared 2 groups of 95 rectal cancer patients treated preoperatively with either short-course (45 patients) or long-course radiotherapy (50 patients). Interestingly, in the non-neoplastic mucosa we identified features closely mimicking dysplasia/pre-neoplasia only in the group treated with short-course radiotherapy. Pathologists awareness of radiation-induced abnormalities is essential, as the misinterpretation may lead to patient’s overtreatment. In our study, next generation sequencing analysis supported the morphological concept that short-course radiotherapy-induced abnormalities do not represent true dysplasia, as somatic mutations were not identified in “dysplastic-like” tissues.

**Abstract:**

Preoperative radiotherapy is a widely accepted treatment procedure in rectal cancer. Radiation-induced changes in the tumor are well described, whereas less attention has been given to the non-neoplastic mucosa. Our aim is to provide a detailed analysis of the morphological features present in non-neoplastic mucosa that pathologists need to be familiar with, in order to avoid misdiagnosis, when evaluating rectal cancer specimens of patients preoperatively treated with radiotherapy, especially with short-course regimen. We compared 2 groups of 95 rectal cancer patients treated preoperatively with either short-course (45 patients) or long-course radiotherapy (50 patients). Depending on the type of protocol, different histopathological features, in terms of inflammation, glandular abnormalities and endocrine differentiation were seen in the non-neoplastic mucosa within the irradiated volume. Of note, features mimicking dysplasia, such as crypt distortion, nuclear and cytoplasmic atypia of glandular epithelium, were identified only in the short-course group. DNA mutation analysis, using a panel of 56 genes frequently mutated in cancer, and p53 immunostaining were performed on both tumor and radiation-damaged mucosa in a subset of short course cases. Somatic mutations were identified only in tumors, supporting the concept that tissues with radiation-induced “dysplastic-like” features are not genetically transformed. Pathologists should be aware of the characteristic morphological changes induced by radiation. The presence of features simulating dysplasia in the group treated with short-course radiotherapy may lead to serious diagnostic mistakes, if erroneously interpreted. Next generation sequencing (NGS) analysis further validated the morphological concept that radiation-induced abnormalities do not represent pre-neoplastic lesions.

## 1. Introduction

Rectal cancer is one of the most common malignant tumors in western countries [1,2]. Radiotherapy represents the standard treatment for locally advanced rectal cancer with a high risk of local recurrence, if treated with surgery alone. Two neoadjuvant regimens are accepted: preoperative long-course radiotherapy (PLRT) with concomitant chemotherapy and preoperative short-course radiotherapy (PSRT). PSRT is recommended for tumors considered amenable to surgery. PLRT is preferred for either low-seated or bulky, unresectable tumors which should benefit from radiation-induced down staging [3,4,5,6,7,8]. PLRT protocol consists of 45–50 gray (Gy) in 4–6 weeks, followed by surgery four weeks later. PSRT consists of 25 Gy administered in five consecutive days, followed by surgery a few days after. Preoperative radiation may produce tumor regression by replacing neoplastic glands with fibrosis and inflammation. Tumor regression is mainly seen in PLRT [9,10]. PSRT is not associated with significant tumor regression, as the interval from the end of radiotherapy to surgery is too short to allow tumor down-staging.

Very few studies analyzed in detail the histopathological features of radiation damage on normal colonic mucosa [11,12]. Depending on the type of preoperative radiotherapy, different modifications may occur in normal colonic mucosa in terms of inflammation and glandular abnormalities [12]. Intrigued by the observation that PSRT-associated morphological abnormalities may simulate dysplasia, causing possible diagnostic misinterpretation, we designed the current study comparing two groups of rectal cancer patients treated either with PSRT or PLRT. The “dysplastic-like” features in irradiated normal mucosa were observed only in PSRT specimens. To confirm that “dysplastic-like” changes were not true dysplasia, we performed DNA mutation analysis of both tumor and mucosa with atypia, on a subset of PSRT cases. Somatic mutations were present only in tumors, suggesting that tissues with radiation-induced “dysplastic-like” features are not genetically transformed. As immunohistochemical p53 staining is considered a surrogate for mutational analysis [13,14,15], we performed p53 immunohistochemistry (IHC) in the same subset of PSRT cases evaluated by next generation sequencing (NGS) analysis. Additionally, we assessed the presence of endocrine elements in benign and malignant tissue of both PSRT and PLRT cases.

## 2. Results

The clinicopathological features of PSRT and PLRT cases are summarized in Table 1.

### 2.1. Tumor Regression

According to the tumor regression system by Dworak et al. [16], tumors were classified as not regressed (Grade 0) or with regression from Grade 1 to Grade 4. In the PSRT group, 31 cases were classified as not regressed (Grade 0) and 14 cases as Grade 1. In the PLRT group the tumors were classified as follows: 12 cases Grade 0; 15 cases Grade 1; 20 cases Grade 2; 2 cases Grade 3 and 1 case Grade 4.

### 2.2. Radiation-Induced Morphological Features

The radiation-induced morphological features were noted in non-neoplastic mucosa samples taken within the irradiated volume. They were identified in the resection margins, if within the irradiated volume. Two types of parameters were analyzed: the inflammatory component and the glandular (“dysplastic-like”) abnormalities.

### 2.3. Inflammatory Component

**Figure 1:**
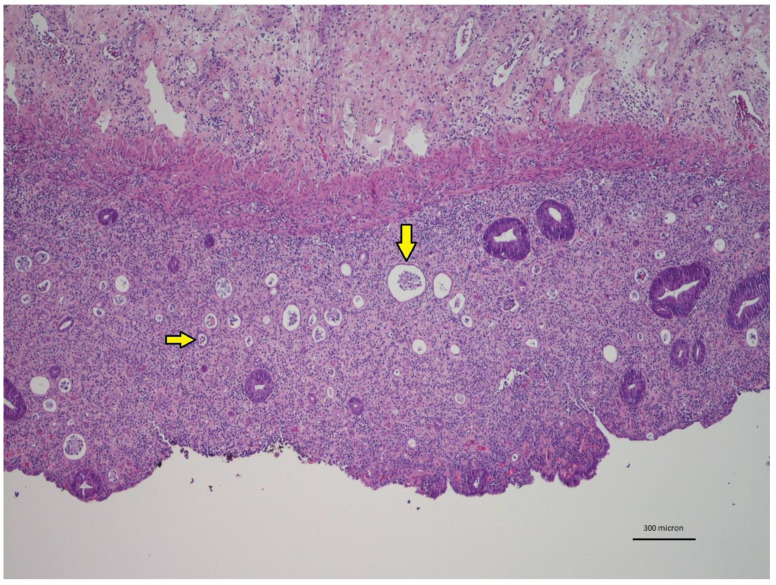
Low power view of radiation-damaged non-neoplastic mucosa in PSRT sample: The lamina propria looks expanded by inflammatory cells and crypts (yellow arrows) are decreased (Hematoxylin and Eosin (HE) 40 times magnified). Scale Bar: 300 microns.

**Figure 2:**
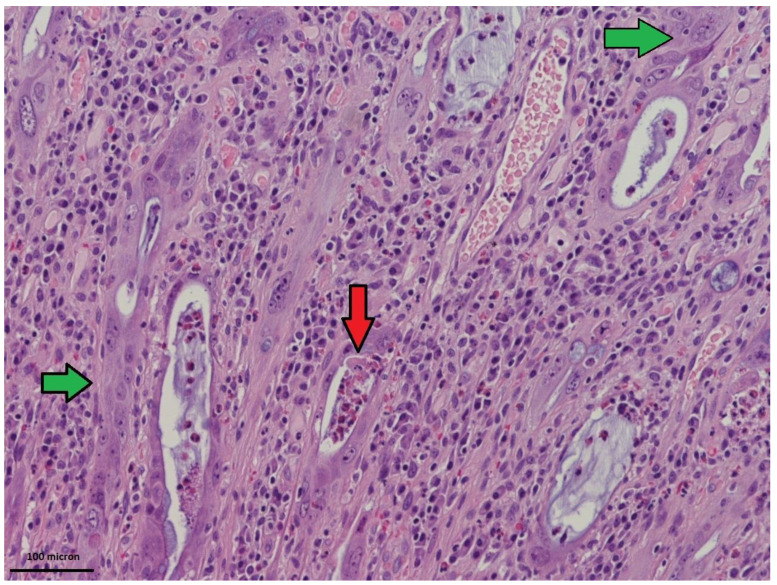
High power view of radiation-damaged non-neoplastic mucosa in PSRT sample: Distorted glands lined by atypical epithelium (green arrows) and eosinophils aggregates (red arrow) within glandular epithelium (Hematoxylin and Eosin (HE) 200 times magnified). Scale Bar: 100 microns.

In PSRT cases, the non-neoplastic mucosa within the irradiated volume showed a moderate to marked inflammation within the lamina propria (Figure 1). The inflammation consisted of histiocytes, lymphocytes, plasma cells and typically numerous eosinophils. The eosinophils were identified in the lamina propria either scattered or in small aggregates and within the glandular epithelium (Figure 2). In PLRT samples the inflammation went from absent to a mild chronic inflammatory infiltrate. Eosinophils were rare.

### 2.4. “Dysplastic-Like” Features

**Figure 3:**
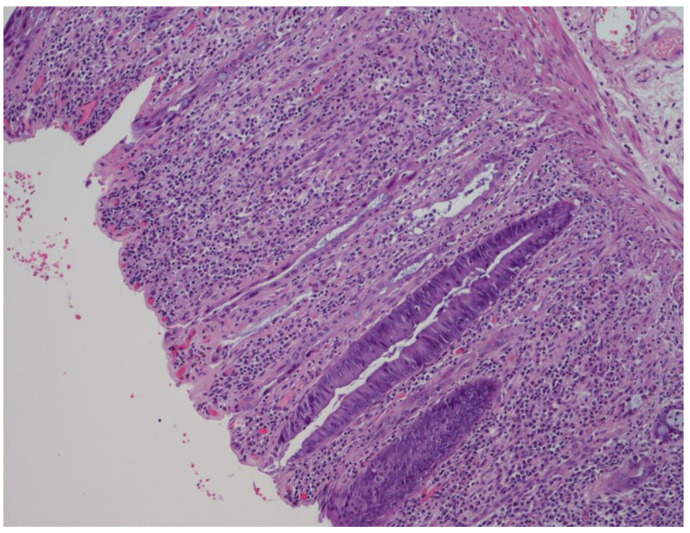
“Dysplastic-like” features in radiation-damaged non-neoplastic mucosa in PSRT sample: glands with slit-like lumen and flattened epithelium close to glands with pseudostratified epithelium (Hematoxylin and Eosin (HE) 200 times magnified).

**Figure 4:**
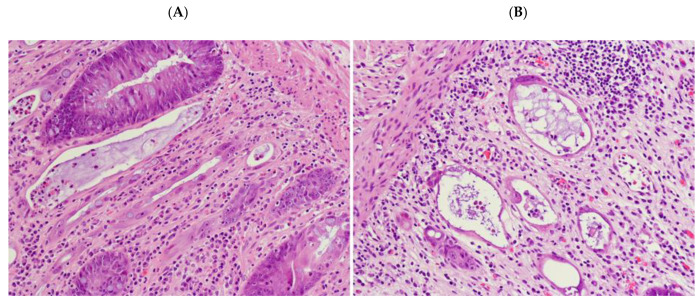
(**A**) “Dysplastic-like” features in radiation-damaged non-neoplastic mucosa in PSRT sample: glands lined by pseudostratified epithelium close to dilated glands with flat epithelium; (**B**) non-neoplastic mucosa in PSRT samples showing dilated glands with flat and atypical epithelial layer (Both A and B are Hematoxylin and Eosin (HE) 200 times magnified).

In PSRT cases, there was a moderate to marked degree of glandular disarray/distortion as well as nuclear and cytoplasmic atypia. The crypts were decreased in number, dilated or with slit-like lumen (Figure 3). The crypt epithelium was either flattened or pseudostratified showing a variable degree of nuclear pleomorphism (Figure 4A,B). The cytoplasm of the crypt epithelium was eosinophilic or vacuolated. Apoptotic bodies were identified. All these features were named “dysplastic-like” for simplicity.

In contrast to the short-course group, in PLRT cases the “dysplastic-like” features were either absent or occasionally identified.

### 2.5. Endocrine Features

The presence of endocrine cells showed differences according to the type of protocol (Table 2).

**Figure 5:**
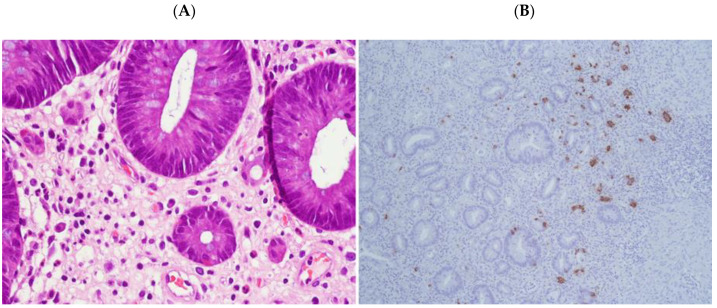
(**A**) Endocrine cells in radiation-damaged non-neoplastic mucosa in PSRT sample (Hematoxylin and Eosin (HE) 200 times magnified); (**B**) chromogranin immunostaining highlighting endocrine cells in radiation-damaged non-neoplastic mucosa in PSRT sample (100 times magnified).

In PSRT cases, the radiation-damaged mucosa showed an increase in endocrine cells (Figure 5A,B) either with isolated cells (5 cases) or micronests (19 cases), absence of endocrine cells was seen in one case. In the PLRT group, the non-neoplastic mucosa within the irradiated volume showed mainly absence of endocrine cells (20 cases) and, more rarely, isolated cells (5 cases). No relevant differences were seen in terms of endocrine differentiation in tumor samples of both protocols.

### 2.6. p53 Immunohistochemical Results

In a subset of 22 PSRT cases, different p53 staining patterns were identified in tumors (Table 3).

**Figure 6:**
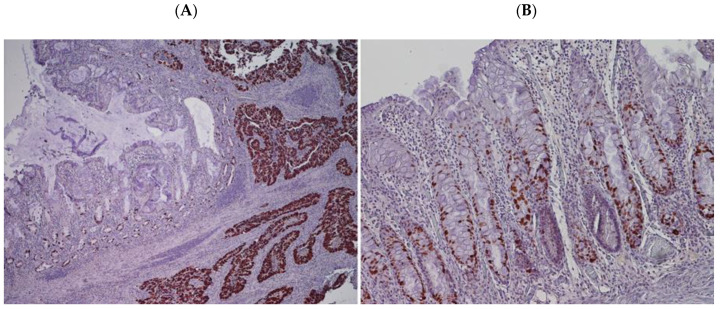
(**A**) “Positive-pattern” of p53 in PSRT tumor sample (right). Few scattered p53-positive cells in mucosa with acute radiation-damage (left) (p53 immunostaining) (100 times magnified); (**B**) Scattered p53-positive cells in the deep portion of glandular epithelium of radiation-damaged non-neoplastic mucosa in PSRT (p53 immunostaining) (200 times magnified).

A strong and diffuse p53 expression (“positive-pattern”) was present in 15/22 tumors (Figure 6A); a complete lack of expression (“negative-pattern”) was seen in 5/22 tumors; scattered p53-positive cells (“reactive-pattern”) were present in 2/22 tumors.

In all samples examined, the mucosa with acute radiation-damage (data not shown in Table 3) showed a positive p53 staining limited to the deep portion of the glandular epithelium which represents the proliferative compartment of the glands (Figure 6B).

### 2.7. Genetic Alterations in Tumors and in Tissue Samples with “Dysplastic-Like” Features

**Figure 7:**
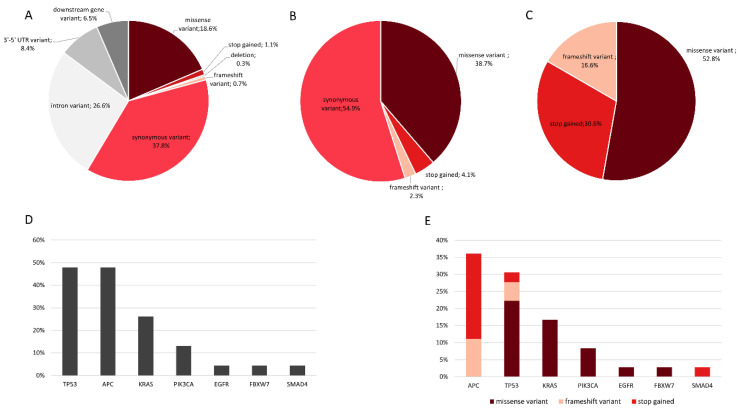
Mutational Profile of 44 tumors and mucosa tissues from 22 PSRT patients: (**A**) distribution of 958 genetic alterations according with position and functional effects; (**B**) distribution of 266 coding region variants according with predicted functional effects; (**C**) distribution of 36 tumor-associated somatic mutations according with predicted functional effects; (**D**) frequency distribution of somatic mutated genes in analysed patients; (**E**) frequency distribution of somatic variants with different functional effect in mutated genes.

**Figure 8:**
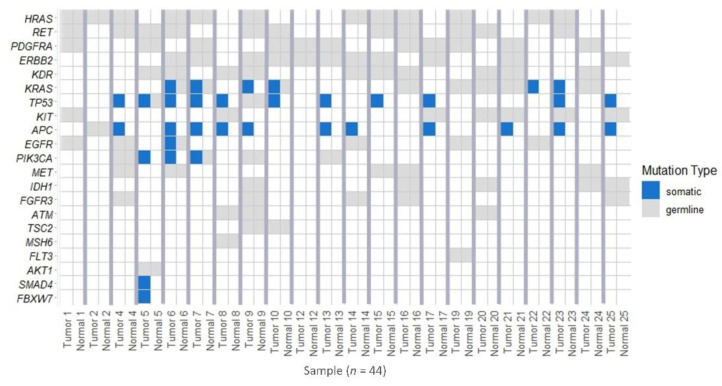
Waterfall plot representing the gene variants occurred in each sample. Blue squares indicate somatic variants, grey squares indicate germinal variant. Tumor and radiation-damaged mucosa tissue from the same patient are side by side.

We evaluated quality of 48 tissue DNA from both tumor and “dysplastic-like” mucosa of 24 PSRT patients included in the study. Only in 22 patients we obtained, from both components, DNA eligible for NGS analysis. On these samples, we performed a deep sequencing analysis on a commercial panel of 56 genes frequently mutated in cancer, detecting 958 alterations (Figure 7A). Subsequently, the analysis was restricted to coding regions variants, excluding intronic, 5′-3′ UTR and downstream gene variants. 266 mutations were found, of which 146 (54.9%) were synonymous, 103 (38.7%) missense and a small percentage was composed by stop gained and frameshift alterations (4.1% and 2.3% respectively) (Figure 7B). Based on literature, Exome Aggregation Consortium (ExAc) frequency and variant frequency 230/266 alterations (86.5%) were classified as germinal and 36/266 (13.5%) as somatic. In each patient, “dysplastic-like” mucosa and tumor, shared the same germline alterations confirming the constitutiveness of these variants and the validity of our analysis (Table S1). By contrast, somatic mutations were present only in tumors, suggesting that “dysplastic-like” tissues are not genetically transformed (Figure 8). Between tumor-associated somatic mutations 52.8% were missense, 30.6% were stop gained and 16.6% were frameshift (Figure 7C).

Somatic mutations were detected in 7/56 genes and gene alterations frequencies were in line with The Cancer Genome Atlas (TCGA (https://www.cancer.gov/about-nci/organization/ccg/research/structural-genomics/tcga)) data on colorectal cancer (CRC). The most frequently mutated genes were *APC* and *TP53* detected in 47.8% of patients. 66.7% of the described somatic alterations were annotated in these genes, mutations in APC were in particular stop gained and frameshift variants while *TP53* presented a majority of missense mutations. Moreover, 26.1% of analyzed tumors presented missense mutations in *KRAS* (6/22) and 13% in *PIK3CA*. Finally, only one tumor presented a missense mutation in *FBXW7* and a stop mutation in *SMAD4* and one had a missense mutation in *EGFR* (Figure 7D,E).

### 2.8. Comparison between P53 Phenotype and TP53 Genotype in Tumor and Mucosa with “Dysplastic-Like” Features

Mutant *TP53* was associated with diffuse and intense p53 immunostaining (“positive-pattern”) in 9/22 tumors. This “positive-pattern” was also present in 6/22 tumors with wild-type *TP53*. Of 5/22 completely p53-negative (“negative-pattern”) tumors, 2 cases had a mutation of *TP53* and 3 cases were wild-type *TP53*. Two tumors with wild-type *TP53* showed only rare, scattered p53-positive cells (“reactive-pattern”).

All 22 samples of mucosa with “dysplastic-like” features were *TP53* wild-type and showed p53 immunostaining only in the deep, proliferative portion of the glandular epithelium.

## 3. Discussion

Preoperative radiotherapy is used increasingly in the management of rectal cancer patients. It is essential for pathologists to be familiar with radiation-induced morphological modifications. Radiation-induced changes in the tumor are well described, particularly tumor down-staging as a consequence of long-term radiotherapy [16]. Less attention has been given to the non-neoplastic mucosa.

The chronic radiation colitis pattern (dilated capillaries within hyalinized lamina propria), identified months or years after radiotherapy, is well known by pathologists [17]. The acute radiation colitis histology is occasionally described [12].

Intrigued by the observation that PSRT-associated epithelial changes simulate dysplasia, we designed the current study comparing PLRT cases with PSRT ones. The short time interval between the end of radiotherapy and surgery is the reason why in the short-term group we found acute radiation colitis features. (i.e., acute inflammation rich in eosinophils, crypt distortion, epithelial atypia, apoptotic bodies). These changes were restricted to the mucosa included in the irradiated volume. “Dysplastic-like” features in irradiated normal mucosa were observed only in PSRT specimens. In esophagus carcinoma Brien et al. [18] noted that radiation-induced atypia within benign glands mimics dysplasia or even residual carcinoma. The expanding use of short-course radiotherapy leads the pathologists to have to evaluate acute radiation colitis and its differential. The misinterpretation of acute radiation colitis as dysplasia is a significant diagnostic error. When frozen sections are performed on resection margins, an erroneous diagnosis of dysplasia can cause patient overtreatment.

CRC is the result of accumulation of multiple genetic and epigenetic aberrations [19]. CRC begins as a benign adenomatous intestinal polyp, evolving to adenoma with high grade dysplasia, invasive adenocarcinoma and metastatic disease [19]. According to the multistep genetic model by Fearon and Vogelstein [20], the APC (adenomatous polyposis) mutation is the first event transforming normal colorectal epithelium to adenoma. APC inactivation is followed by oncogenic *KRAS* mutations in the adenomatous stage and eventually chromosome 18q deletion and inactivation of tumor-suppressor gene *TP53* on chromosome 17p in the transition to malignancy.

In a subset of PSRT cases, we performed NGS analysis on both tumor and irradiated mucosa with “dysplastic-like” features. Somatic mutations were found only in tumor samples. The most frequently mutated genes were *TP53* and *APC*, consistently with the literature data reporting *APC* as the most frequently found mutation in CRC followed by *TP53*. Somatic mutations were not identified in mucosa with acute radiation colitis changes, supporting the concept that tissues with features mimicking dysplasia were not genetically transformed.

Consistently with previous studies [14,15], p53 overexpression (positive-pattern) was found to closely correlate with *TP53* mutation, as in most tumors with mutated *TP53* (9/11; 81.8%) a diffuse and intense p53 staining was present. This positive pattern is generally considered indicative of a missense *TP53* mutation. As expected, a complete absence of nuclear staining (negative-pattern) was identified in 2 *TP53* mutated tumors (18.1%) presenting a stop gained or frameshift variant responsible for a protein loss of function [14,15]. Non-concordant data were obtained only in 6 of the investigated samples. This is not surprising being already reported in literature that IHC and NGS may sometimes result in divergent conclusions. While a formal explanation for this it has not yet been provided, this discrepancy is likely associated with p53 alterations, like copy number variation (CNV), which cannot be evaluated by the employed NGS approach.

Interestingly neither the positive- or negative-patterns of p53 staining were seen in the mucosa with acute radiation damage. p53 labeling was restricted to the proliferative compartment of the glandular epithelium in the mucosa with “dysplastic-like” features, in keeping with the physiologic activity of p53 protein. Accordingly, the mucosa with radiation-induced atypia was consistently *TP53* wild-type.

An increase in endocrine cells was noted in the irradiated non-neoplastic mucosa in the PSRT group. In analogy to previous observations in esophageal adenocarcinoma after neo-adjuvant treatment [21], we interpreted the endocrine cells as residual normal endocrine elements, appearing more conspicuous for the radiation-induced glandular damage causing “passive clustering” [22]. In support of this observation, no endocrine cells increase was observed in the non-neoplastic irradiated mucosa in PLRT cases, in which acute radiation colitis features were characteristically absent. Unlike previous reports [21], which found the extent of endocrine differentiation within the tumor to be proportional to the degree of tumor regression, we did not observe increase in endocrine cells within the tumor in either group of patients.

## 4. Materials and Methods

### 4.1. Patients Cohort

A retrospective study was performed on surgical resection specimens of 95 patients with rectal adenocarcinoma, treated with radical surgery and preoperative radiotherapy between 2000 and 2017 at the Azienda Unità Sanitaria Locale-IRCCS of Reggio Emilia, Italy.

The patients were divided into two groups according to the preoperative radiation protocol.

The first group consisted of 45 patients (27 males and 18 females; mean age 75.2; age range: from 46 to 90 years) treated with PSRT (duration: 5 consecutive days with a total dose of 25 gray (Gy) in 5 fractions of 5 Gy daily) with immediate surgery within 8–10 days after the end of radiation.

The second group of 50 patients (25 males and 25 females; mean age 62.4; age range: from 38 to 79 years) included unresectable or very low-seated neoplasms. The latter group received PLRT (duration of radiotherapy: 4–6 weeks, with a total dose of 45–50 Gy fractioned in single doses of 1.8–2 Gy daily) with surgery 4–6 weeks later.

In both protocols contrasted computerized tomography (CT) scan of lower abdomen and pelvis was performed. CT scan images were transferred to the pretreatment planning system for contouring the target volume and organs at risk. The gross total volume (GTV) was contoured based on clinical data, endoscopic ultrasound (EUS) and magnetic resonance imaging (MRI). The clinical target volume (CTV) included at least a 3 cm craniocaudal margin to the GTV plus mesorectum, presacral and internal iliac lymph nodes. The external iliac nodes were included for T4 tumors involving anterior structures. PLRT patients were subsequently treated with chemotherapy.

### 4.2. Morphological Examination

All hematoxylin and eosin-stained slides were reviewed independently by two of the authors (D.L., Z.M.). None of the cancers displayed neuroendocrine differentiation before radiotherapy. Non-neoplastic mucosa and tumor sections were assessed to detect radiation-induced changes and endocrine cells presence. Representative tumor sections were selected for grading of tumor regression according to Dworak et al. [16] system in 5 points as follows: grade 0—no regression; grade 1—dominant tumor mass with obvious fibrosis and/or vasculopathy; grade 2—dominantly fibrotic changes with few tumor cells or groups; grade 3—very few tumor cells in fibrotic tissue with or without mucous substance; grade 4—no tumor cells, only fibrotic mass (total regression).

The degree of radiation damage was assessed in the non-neoplastic mucosa, including the surgical resection margins. The following histological parameters were evaluated: inflammation in the lamina propria, architectural crypt distortion, nuclear and cytoplasmic atypia of glandular epithelium and apoptotic bodies. All these features were categorized as being present or absent and, if present, semi-quantitatively graded as mild, moderate and severe.

### 4.3. IHC Analysis

In 25 PSRT and 25 PLRT cases, the presence of endocrine features was assessed morphologically and with chromogranin A (LK 2H10, monoclonal antibody, Ventana, Oro Valley, AZ, USA) and synaptophysin (SP11, monoclonal antibody, Ventana, Oro Valley, AZ, USA) immunostains in both tumor and radiation-damaged non-neoplastic mucosa.

The endocrine differentiation was scored as follows: absent, isolated endocrine cells, endocrine cells micronests. Endocrine micronests were defined as 5–15 cells clusters.

In a subset of 22 PSRT patients (the same subset in which samples were eligible for genomic DNA analysis), p53 (DO-7 monoclonal antibody, Ventana, Oro Valley, AZ, USA) immunohistochemical expression was evaluated in both the tumor and the mucosa with radiation damage.

### 4.4. NGS Mutational Analysis

Genomic DNA, from both tumor and “dysplastic-like” mucosa samples of a subset of 24 PSRT patients was isolated by Maxwell DNA FFPE Kit (Promega, Madison, WI, USA), according to the manufacturer instructions. DNA concentration was determined using Qubit dsDNA HS assay kit (Invitrogen, Carlsbad, CA, USA). Twenty-two samples were eligible for sequencing analysis. For the NGS analysis, DNA libraries were prepared using Myriapod NGS-IL 56G Onco Panel for Illumina (Diatech Pharmacogenetics, Jesi, Italy), that allows the identification of main mutations in 56 oncogenes, following the manufacturer instructions. Libraries quality and quantity were assessed by Agilent Bioanalyzer High Sensitivity kit (Agilent Technologies, Santa Clara, CA, USA) and Qubit dsDNA HS assay kit (Invitrogen, Carlsbad, CA, USA) respectively. Sequencing run on Illumina MiSeq V2 (2x151) cartridge (Illumina, San Diego, CA, USA). Sequencing data analysis was conducted by Myriapod NGS Analysis software (v 4.0.2) and further analysis were performed using R software (v 3.5.1).

## 5. Conclusions

The present data are a comprehensive morphological description of radiation-induced abnormalities after preoperative radiotherapy for rectal cancer. NGS analysis supported the morphological concept that PSRT-induced “dysplastic-like” tissues are not genetically transformed. Short-course radiotherapy may induce morphological features closely simulating dysplasia. The misinterpretation may lead to patient’s overtreatment. When facing rectal cancer specimens, pathologists need the complete patient’s clinical history and must ask if preoperative radiotherapy has been given. p53 immunostaining may be of help in problematic cases.

## Figures and Tables

**Table 1:** Clinicopathological features of preoperative short-course radiotherapy (PSRT) and preoperative long-course radiotherapy (PLRT) cases.

Clinicopathological Features	PSRT	%	PLRT	%	*p* Value
	45	47.4%	50	52.6%	
Age	75.2 years		62.4 years		<0.001
	(46–90)		(38–79)		
Sex					0.410
Female	18	18.9%	25	26.3%	
Male	27	28.4%	25	26.3%	
Tumor regression system by Dworak et al. [16]					<0.001
Grade 0	31	32.6%	12		
Grade 1	14	14.7%	15	15.8%	
Grade 2	0	0.0%	20	21.1%	
Grade 3	0	0.0%	2	2.1%	
Grade 4	0	0.0%	1	1.1%	
Inflammation					<0.001
absent	4	4.2%	35		
mild	4	4.2%	13	13.7%	
moderate	23	24.2%	1	1.1%	
severe	14	14.7%	1	1.1%	
Architectural crypt distortion					<0.001
absent	4	4.2%	47		
mild	4	4.2%	2	2.1%	
moderate	19	20.0%	1	1.1%	
severe	18	18.9%	0	0.0%	
Nuclear and cytoplasmic atypia of glandular epithelium					<0.001
absent	4	4.2%	49		
mild	3	3.2%	1	1.1%	
	20	21.1%	0	0.0%	
	18	18.9%	0	0.0%	
Apoptotic bodies					<0.001
	5	5.3%	49		
	40	42.1%	1	1.1%	

PSRT: preoperative short-course radiotherapy; PLRT: preoperative long-course radiotherapy.

**Table 2:** Endocrine features in PSRT and PLRT cases.

Endocrine Features	PSRT		PLRT		*p* Value
	N°	%	N°	%	
	25	50.0%	25	50.0%	
Endocrine differentiation in radiation-damaged mucosa					<0.001
absent	1	2.0%	20	40.0%	
isolated endocrine cells	5	10.0%	5	10.0%	
endocrine cells micronests	19	38.0%	0	0.0%	
Endocrine differentiation in tumor					0.490
absent	23	46.0%	25	50.0%	
isolated endocrine cells	1	2.0%	0	0.0%	
endocrine cells micronests	1	2.0%	0	0.0%	

PSRT: preoperative short-course radiotherapy; PLRT: preoperative long-course radiotherapy; N°: number of cases.

**Table 3:** Comparative data between p53 phenotype and TP53 genotype in PSRT tumor samples.

p53 IHC	TP53 NGS		Total of Cases
	MUT	WT	
Negative-pattern	2	3	5
Positive-pattern	9	6	15
Reactive-pattern	0	2	2
Total of cases	11	11	22

IHC: immunohistochemistry; NGS: next generation sequencing; MUT: mutated; WT: wild-type.

## Supplementary Materials

The following are available online at https://www.mdpi.com/2072-6694/12/9/2571/s1, Table S1: List of genetic variants detected in tumor and normal tissue of each patient.
