# Supplementary material for: Acute Radiation Colitis after Preoperative Short-Course Radiotherapy for Rectal Cancer: A Morphological, Immunohistochemical and Genetic Study
Source: Cancers (Basel). 2020 Sep 9;12(9):2571. doi: 10.3390/cancers12092571 (PMC7563849; doi:10.3390/cancers12092571)
Supplement: Supplementary file 1 [file cancers-12-02571-s001.pdf]

Supplementary Materials: Acute Radiation Colitis After Preoperative Short-Course Radiotherapy for Rectal Cancer: A Morphological, Immunohistochemical and Genetic Study

Magda Zanelli, Alessia Ciarrocchi, Giovanni De Petris, Maurizio Zizzo, Massimo Costantini, Alessandra Bisagni, Federica Torricelli, Davide Nicoli, Dafne Ramundo, Stefano Ricci, Andrea Palicelli, Francesca Sanguedolce, Stefano Ascani, Carolina Castro Ruiz, Valerio Annessi, Raffaella Zamponi, Mara Bortesi, Veronica Martino, Marialisa Marchetti and Loredana De Marco

Table 1. List of genetic variants detected in tumor and normal tissue of each patient.

	Tumor		Normal			
Patient	Gene	AA Change	Gene	AA Change	Coding Effect	Origin
Patient 1	*EGFR*	p.Ala613Ala	*EGFR*	p.Ala613Ala	synonymous_variant	germline
	HRAS	p.His27His	*HRAS*	p.His27His	synonymous_variant	germline
	KIT	p.Met541Leu	*KIT*	p.Leu862Leu	missense_variant	germline
	KIT	p.Leu862Leu	*KIT*	p.Met541Leu	synonymous_variant	germline
	PDGFRA	p.Val824Val	*PDGFRA*	p.Val824Val	synonymous_variant	germline
	RET	p.Ser904Ser	*RET*	p.Ser904Ser	synonymous_variant	germline
Patient 2	*APC*	p.Ile1307Lys	*APC*	p.Ile1307Lys	missense_variant	germline
	HRAS	p.His27His	*HRAS*	p.His27His	synonymous_variant	germline
Patient 4	*EGFR*	p.Gln787Gln	*EGFR*	p.Gln787Gln	synonymous_variant	germline
	FGFR3	p.Phe384Leu	*FGFR3*	p.Phe384Leu	missense_variant	germline
	HRAS	p.His27His	*HRAS*	p.His27His	synonymous_variant	germline
	MET	p.Ser178Ser	*MET*	p.Ser178Ser	synonymous_variant	germline
	PIK3CA	p.Ile391Met	*PIK3CA*	p.Ile391Met	missense_variant	germline
	RET	p.Ser904Ser	*RET*	p.Ser904Ser	synonymous_variant	germline
	APC	p.Arg876*			stop_gained	somatic
	APC	p.Ile1307fs			frameshift_variant	somatic
	TP53	p.Arg273Cys			missense_variant	somatic
Patient 5	*AKT1*	p.Gln43His	*AKT1*	p.Gln43His	missense_variant	germline
	KDR	p.Gln472His	*KDR*	p.Gln472His	missense_variant	germline
	PDGFRA	p.Val824Val	*PDGFRA*	p.Val824Val	synonymous_variant	germline
	RET	p.Ser904Ser	*RET*	p.Ser904Ser	synonymous_variant	germline
	TP53	p.Pro36Pro	*TP53*	p.Pro36Pro	synonymous_variant	germline
	TP53	p.Arg213Arg	*TP53*	p.Arg213Arg	synonymous_variant	germline
	FBXW7	p.Arg347Cys			missense_variant	somatic
	PIK3CA	p.Glu542Lys			missense_variant	somatic
	SMAD4	p.Gln245*			stop_gained	somatic
	TP53	p.Pro152Leu			missense_variant	somatic
Patient 6	*EGFR*	p.Gln787Gln	*EGFR*	p.Gln787Gln	synonymous_variant	germline
	ERBB2	p.Ile655Val	*ERBB2*	p.Ile655Val	missense_variant	germline
	HRAS	p.His27His	*HRAS*	p.His27His	synonymous_variant	germline
	KDR	p.Gln472His	*KDR*	p.Gln472His	missense_variant	germline
	KRAS	p.Asp173Asp	*KRAS*	p.Asp173Asp	synonymous_variant	germline
	MET	p.Arg970Cys	*MET*	p.Arg970Cys	missense_variant	germline
	RET	p.Ser904Ser	*RET*	p.Ser904Ser	synonymous_variant	germline
	APC	p.Glu1379*			stop_gained	somatic
	EGFR	p.Asp855Gly			missense_variant	somatic
	KRAS	p.Gly12Val			missense_variant	somatic
	PIK3CA	p.Glu542Val			missense_variant	somatic
	TP53	p.His214Arg			missense_variant	somatic
Patient 7	*ERBB2*	p.Ile655Val	*ERBB2*	p.Ile655Val	missense_variant	germline
	HRAS	p.His27His	*HRAS*	p.His27His	synonymous_variant	germline
	KRAS	p.Asp173Asp	*KRAS*	p.Asp173Asp	synonymous_variant	germline
	PDGFRA	p.Val824Val	*PDGFRA*	p.Val824Val	synonymous_variant	germline
	PIK3CA	p.Ile391Met	*PIK3CA*	p.Ile391Met	missense_variant	germline
	APC	p.Gln1367*			stop_gained	somatic
	KRAS	p.Gly12Asp			missense_variant	somatic
	PIK3CA	p.Glu545Lys			missense_variant	somatic
	TP53	p.Gly293fs			frameshift_variant	somatic
Patient 8	*ATM*	p.Pro604Ser	*ATM*	p.Pro604Ser	missense_variant	germline
	KDR	p.Val297Ile	*KDR*	p.Val297Ile	missense_variant	germline
	KIT	p.Lys546Lys	*KIT*	p.Lys546Lys	synonymous_variant	germline

	MSH6	p.Gly1072Val	*MSH6*	p.Gly1072Val	missense_variant	germline
	APC	p.Thr1301fs			frameshift_variant	somatic
	TP53	p.Arg175His			missense_variant	somatic
Patient 9	*ATM*	p.Phe858Leu	*ATM*	p.Phe858Leu	missense_variant	germline
	ERBB2	p.Ile655Val	*ERBB2*	p.Ile655Val	missense_variant	germline
	FGFR3	p.Asn294Asn	*FGFR3*	p.Asn294Asn	synonymous_variant	germline
	HRAS	p.His27His	*HRAS*	p.His27His	synonymous_variant	germline
	IDH1	p.Gly105Gly	*IDH1*	p.Gly105Gly	synonymous_variant	germline
	KDR	p.Cys482Arg	*KDR*	p.Cys482Arg	missense_variant	germline
	KDR	p.Gln472His	*KDR*	p.Gln472His	missense_variant	germline
	PIK3CA	p.Ile391Met	*PIK3CA*	p.Ile391Met	missense_variant	germline
	RET	p.Ser904Ser	*RET*	p.Ser904Ser	synonymous_variant	germline
	TP53	p.Arg213Arg	*TP53*	p.Arg213Arg	synonymous_variant	germline
	TSC2	p.Ser1557Ser	*TSC2*	p.Ser1557Ser	synonymous_variant	germline
	APC	p.Arg1114*			stop_gained	somatic
	KRAS	p.Gly12Val			missense_variant	somatic
Patient 10	*ERBB2*	p.Ile655Val	*ERBB2*	p.Ile655Val	missense_variant	germline
	KRAS	p.Asp173Asp	*KRAS*	p.Asp173Asp	synonymous_variant	germline
	PDGFRA	p.Val824Val	*PDGFRA*	p.Val824Val	synonymous_variant	germline
	RET	p.Ser904Ser	*RET*	p.Ser904Ser	synonymous_variant	germline
	TSC2	p.Ala1560Ala	*TSC2*	p.Ala1560Ala	synonymous_variant	germline
	KRAS	p.Gly12Ala			missense_variant	somatic
	TP53	p.Arg306*			stop_gained	somatic
Patient 12	*PDGFRA*	p.Val824Val	*PDGFRA*	p.Val824Val	synonymous_variant	germline
	ERBB2	p.Ile655Val	*ERBB2*	p.Ile655Val	missense_variant	germline
Patient 13	*ERBB2*	p.Ile655Val	*ERBB2*	p.Ile655Val	missense_variant	germline
	PDGFRA	p.Val824Val	*PDGFRA*	p.Val824Val	synonymous_variant	germline
	PIK3CA	p.Ile391Met	*PIK3CA*	p.Ile391Met	missense_variant	germline
	APC	p.Glu1309fs			frameshift_variant	somatic
	TP53	p.Leu257Pro			missense_variant	somatic
Patient 14	*EGFR*	p.Gln787Gln	*EGFR*	p.Gln787Gln	synonymous_variant	germline
	ERBB2	p.Leu696Leu	*ERBB2*	p.Leu696Leu	synonymous_variant	germline
	FGFR3	p.Asn294Asn	*FGFR3*	p.Asn294Asn	synonymous_variant	germline
	HRAS	p.His27His	*HRAS*	p.His27His	synonymous_variant	germline
	KDR	p.Val297Ile	*KDR*	p.Val297Ile	missense_variant	germline
	APC	p.Arg1114*			stop_gained	somatic
Patient 15	*ERBB2*	p.Ile655Val	*ERBB2*	p.Ile655Val	missense_variant	germline
	HRAS	p.His27His	*HRAS*	p.His27His	synonymous_variant	germline
	KDR	p.Gln472His	*KDR*	p.Gln472His	missense_variant	germline
	KDR	p.Val297Ile	*KDR*	p.Val297Ile	missense_variant	germline
	MET	p.Ser178Ser	*MET*	p.Ser178Ser	synonymous_variant	germline
	MET	p.Asn375Ser	*MET*	p.Asn375Ser	missense_variant	germline
	RET	p.Ser904Ser	*RET*	p.Ser904Ser	synonymous_variant	germline
	TP53	p.Val272Leu			missense_variant	somatic
Patient 16	*FGFR3*	p.Asn294Asn	*FGFR3*	p.Asn294Asn	synonymous_variant	germline
	HRAS	p.His27His	*HRAS*	p.His27His	synonymous_variant	germline
	IDH1	p.Gly105Gly	*IDH1*	p.Gly105Gly	synonymous_variant	germline
	KDR	p.Val297Ile	*KDR*	p.Val297Ile	missense_variant	germline
	KRAS	p.Asp173Asp	*KRAS*	p.Asp173Asp	synonymous_variant	germline
	MET	p.Arg970Cys	*MET*	p.Arg970Cys	missense_variant	germline
	PDGFRA	p.Val824Val	*PDGFRA*	p.Val824Val	synonymous_variant	germline
	RET	p.Ser904Ser	*RET*	p.Ser904Ser	synonymous_variant	germline
Patient 17	*ERBB2*	p.Ile655Val	*ERBB2*	p.Ile655Val	missense_variant	germline
	KIT	p.Lys546Lys	*KIT*	p.Lys546Lys	synonymous_variant	germline
	PDGFRA	p.Val824Val	*PDGFRA*	p.Val824Val	synonymous_variant	germline
	APC	p.Ser1356*			stop_gained	somatic
	TP53	p.Leu188fs			frameshift_variant	somatic
Patient 19	*EGFR*	p.Ala613Ala	*EGFR*	p.Ala613Ala	synonymous_variant	germline
	FLT3	p.Ile827Leu	*FLT3*	p.Ile827Leu	missense_variant	germline
	HRAS	p.His27His	*HRAS*	p.His27His	synonymous_variant	germline
	KRAS	p.Asp173Asp	*KRAS*	p.Asp173Asp	synonymous_variant	germline
	PDGFRA	p.Val824Val	*PDGFRA*	p.Val824Val	synonymous_variant	germline
	RET	p.Ser904Ser	*RET*	p.Ser904Ser	synonymous_variant	germline
Patient 20	*ATM*	p.Asp2661Asp	*ATM*	p.Asp2661Asp	synonymous_variant	germline
	IDH1	p.Gly105Gly	*IDH1*	p.Gly105Gly	synonymous_variant	germline
	KDR	p.Gln472His	*KDR*	p.Gln472His	missense_variant	germline
	KIT	p.Met541Leu	*KIT*	p.Leu862Leu	missense_variant	germline
	KIT	p.Leu862Leu	*KIT*	p.Met541Leu	synonymous_variant	germline
	KRAS	p.Asp173Asp	*KRAS*	p.Asp173Asp	synonymous_variant	germline
	RET	p.Ser904Ser	*RET*	p.Ser904Ser	synonymous_variant	germline
Patient 21	*PDGFRA*	p.Val824Val	*PDGFRA*	p.Val824Val	synonymous_variant	germline
	KIT	p.Leu862Leu	*KIT*	p.Leu862Leu	synonymous_variant	germline
	KDR	p.Val297Ile	*KDR*	p.Val297Ile	missense_variant	germline
	KRAS	p.Asp173Asp	*KRAS*	p.Asp173Asp	synonymous_variant	germline
	ERBB2	p.Ile655Val	*ERBB2*	p.Ile655Val	missense_variant	germline

	APC	p.Gln1367*			stop_gained	somatic
Patient 22	*EGFR*	p.Ala613Ala	*EGFR*	p.Ala613Ala	synonymous_variant	germline
	HRAS	p.His27His	*HRAS*	p.His27His	synonymous_variant	germline
	KRAS	p.Gly12Asp			missense_variant	somatic
Patient 23	*HRAS*	p.His27His	*HRAS*	p.His27His	synonymous_variant	germline
	KIT	p.Met541Leu	*KIT*	p.Met541Leu	missense_variant	germline
	KIT	p.Leu862Leu	*KIT*	p.Leu862Leu	synonymous_variant	germline
	RET	p.Ser904Ser	*RET*	p.Ser904Ser	synonymous_variant	germline
	APC	p.Arg876*			stop_gained	somatic
	APC	p.Gly1466fs			frameshift_variant	somatic
	KRAS	p.Gly13Asp			missense_variant	somatic
	TP53	p.Glu258Asp			missense_variant	somatic
Patient 24	*IDH1*	p.Gly105Gly	*IDH1*	p.Gly105Gly	synonymous_variant	germline
	KDR	p.Cys482Arg	*KDR*	p.Cys482Arg	missense_variant	germline
	KDR	p.Gln472His	*KDR*	p.Gln472His	missense_variant	germline
	KRAS	p.Asp173Asp	*KRAS*	p.Asp173Asp	synonymous_variant	germline
	MET	p.Ser178Ser	*MET*	p.Ser178Ser	synonymous_variant	germline
	MET	p.Asn375Ser	*MET*	p.Asn375Ser	missense_variant	germline
	PDGFRA	p.Val824Val	*PDGFRA*	p.Val824Val	synonymous_variant	germline
Patient 25	*ERBB2*	p.Ile655Val	*ERBB2*	p.Ile655Val	missense_variant	germline
	FGFR3	p.Phe384Leu	*FGFR3*	p.Phe384Leu	missense_variant	germline
	IDH1	p.Gly105Gly	*IDH1*	p.Gly105Gly	synonymous_variant	germline
	KIT	p.Lys546Lys	*KIT*	p.Lys546Lys	synonymous_variant	germline
	APC	p.Gln1291*			stop_gained	somatic
	TP53	p.Arg248Gln			missense_variant	somatic

AA: Aminoacids.

© 2020 by the authors. Licensee MDPI, Basel, Switzerland. This article is an open access article distributed under the terms and conditions of the Creative Commons Attribution (CC BY) license (http://creativecommons.org/licenses/by/4.0/).
